# Pancreatic Metastasis of High-Grade Papillary Serous Ovarian Carcinoma Mimicking Primary Pancreas Cancer: A Case Report

**DOI:** 10.1155/2012/943280

**Published:** 2012-07-08

**Authors:** Yusuf Gunay, Ebru Demiralay, Alp Demirag

**Affiliations:** ^1^Department of General Surgery and Abdominal Transplantation, Baskent University Istanbul Hospital, Oymaci Sok. No. 7, Altunizade, 34660 Istanbul, Turkey; ^2^Department of Pathology, Baskent University Istanbul Hospital, Oymaci Sok. No. 7, Altunizade, 34660 Istanbul, Turkey

## Abstract

*Introduction*. Reports of epithelial ovarian carcinomas metastatic to the pancreas are very rare. We herein present a metastasis of high grade papillary serous ovarian cancer to mid portion of pancreas. *Case*. A 42-year-old patient was admitted with a non-specified malignant cystic lesion in midportion of pancreas. She had a history of surgical treatment for papillary serous ovarian adenocarcinoma. A cystic lesion was revealed by an abdominal computerized tomography (CT) performed in her follow up . It was considered as primary mid portion of pancreatic cancer and a distal pancreatectomy was performed. The final pathology showed high-grade papillary serous adenocarcinoma morphologically similar to the previously diagnosed ovarian cancer. *Discussion*. Metastatic pancreatic cancers should be considered in patients who present with a solitary pancreatic mass and had a previous non-pancreatic malignancy. Differential diagnosis of primary pancreatic neoplasm from metastatic malignancy may be very difficult. A biopsy for tissue confirmation is required to differentiate primary and secondary pancreatic tumors. Although, the value of surgical resection is poorly documented, resection may be considered in selected patients. * Conclusion*. Pancreatic metastasis of ovarian papillary serous adenocarcinoma has to be kept in mind when a patient with pancreatic mass has a history of ovarian malignancy.

## 1. Introduction

The pancreas is an uncommon location for a metastasis from other cancers [1, 2]. In a large autopsy series, the prevalence of pancreatic metastasis was described between 6 to 11% [3]. The metastases of pancreas have usually been reported from a primary tumor of the kidney, lung, breast, gastrointestinal tract (stomach, small bowel, and colorectal) or melanoma [4]. Ovarian carcinoma is the most common cause of death from gynecological malignancy in Europe and in the United States [5]. Although the intraperitoneal route of dissemination is considered the most common, ovarian cancer may also metastasize through the lymphatic channels and the hematogenous route [6]. Distant metastases of the ovarian cancer theoretically may occur anywhere; however, pleura, liver, skin, lungs, central nervous system, spleen, bone, and breast are the most common locations [7].

To our best knowledge, pancreatic metastasis of ovarian cancer is uncommon and only few cases have been reported in literature. We here reported a rare metastasis of papillary serous ovarian adenocarcinoma mimicking primary pancreas cancer.

## 2. Case

**Figure 1:**
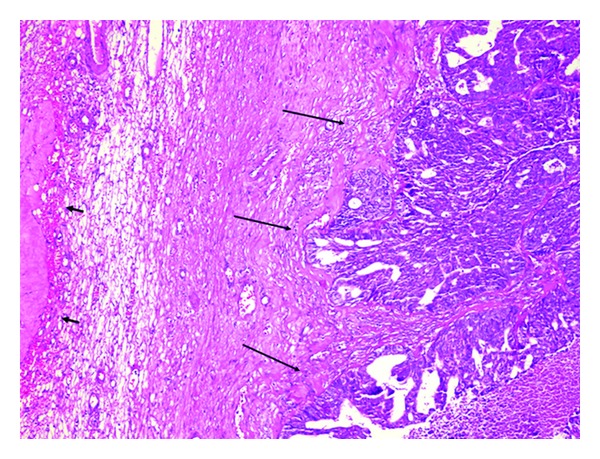
Ovary tissue, in the adjacent of corpus albicans (short arrows), showing irregular neoplastic glandular structure (long arrows), ×200 H & E.

A 42-year-old patient was admitted to our service with a non-specified neoplastic cystic lesion in midportion of pancreas. In her past medical history, she underwent a total abdominal hysterectomy, bilateral salpingooophorectomy, omentectomy, appendectomy, and para-aortic lymph nodes dissection due to papillary serous ovarian adenocarcinoma about ten months prior to current diagnosis (Figure 1). She received chemotherapy following surgery.

**Figure 2:**
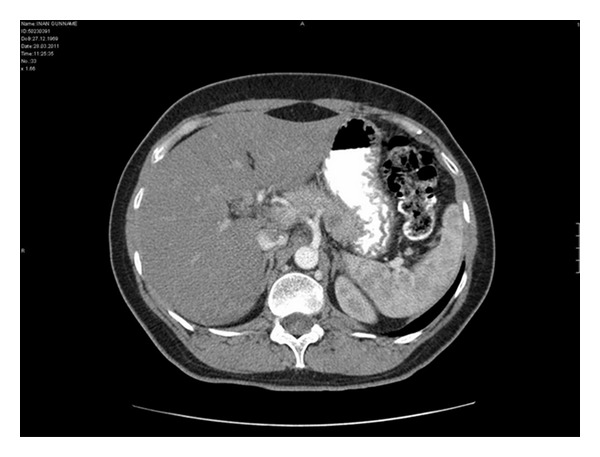
Abdominal CT shows a 4 × 2 cm-sized cystic lesion in mid portion of pancreas.

**Figure 3:**
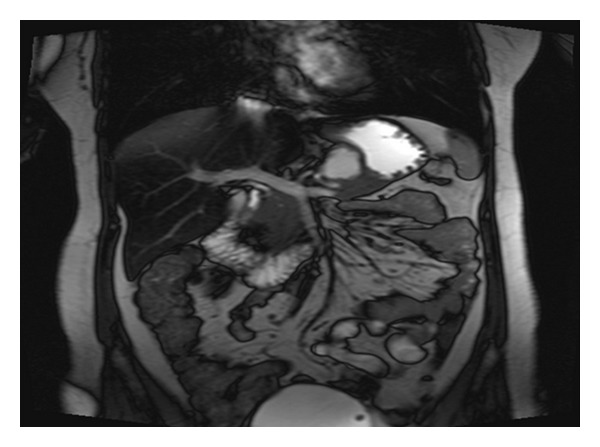
Abdominal MRI reveals a cystic lesion measured 4 × 2 cm in mid portion of pancreas.

The cystic lesion was revealed by an abdominal computerized tomography (CT) and then confirmed by magnetic resonance imaging (MRI) performed regularly in her followup (Figures 2 and 3). To confirm the malignant origin of this lesion, an endoscopic-ultrasound- (EUS-) guided fine needle aspiration (FNA) cytology was performed and the analysis of fluid showed malignant cells, but failed to identify the origin of tumor. The patient was referred to our hospital for further management. Upon admission, physical examination revealed no significant abnormalities except a vague abdominal discomfort. All laboratory tests were unremarkable. 

**Figure 4:**
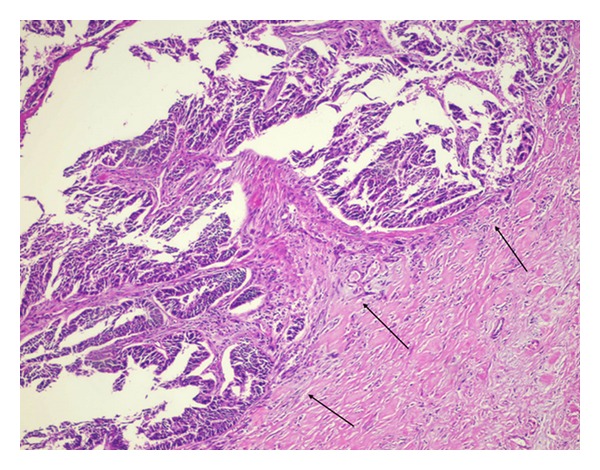
Neoplastic cell islands within fibrotic stroma of pancreas showing similar feature to that of in ovary (arrows), ×200, H & E.

At laparotomy, there was a cystic mass measured approximately 4 × 2 cm localized and confined within midportion of pancreas with no sign of local invasion or distant metastasis. A small amount of fluid from lesion was sent out for cytological evaluation and reported as malignant epithelial neoplastic cell, but failed to identify the origin of tumor. Then it was deemed as primary midportion of pancreatic cancer and a spleen-preserved distal pancreatectomy was performed. The final pathology showed high-grade papillary serous adenocarcinoma morphologically similar to the previously diagnosed ovarian cancer with cancer free of surgical margins (Figure 4). Her postoperative course was uneventful. She received chemotherapy.

## 3. Discussion

Ovarian cancer accounts for approximately 3% of all malignant tumors in women in the USA and ranks second among gynecologic cancers [8]. The diagnosis of serous carcinoma of the ovary is one of the most common diagnoses of epithelial tumors of the ovary [9].

Although the ovarian cancer usually remains confined to the peritoneal cavity at presentation and throughout its course in approximately 85% of the patients [10], distant metastasis is not uncommon. Distant metastases of ovary cancer are present in 8% of patients at the time of diagnosis, and develop in 22% of patients during the course of the disease [11]. They are usually associated to widespread disseminated disease and poor performance status; the effects of these rare metastases are devastating and survival is usually very poor [12]. Significant risk factors for the development of distant metastases were stage, grade, and lymph node involvement. Distant metastases may occur at any time of ovarian cancer throughout its course and the median interval time between diagnosis of ovarian cancer and documentation of distant disease was reported as 15–44 months in the literature [8, 13]. In our case, it was determined ten months following a curative surgical treatment of primary ovarian cancer. The longer survival observed in patients with prolonged interval time between diagnosis of ovarian cancer and detection of distant metastasis probably reflects a biologically indolent course of such tumors [13]. Prognosis of patients who develop distant metastases is poor; however, in selected cases integrated multimodality treatment may result in a palliation of the symptomatology and even in a prolonged survival [11].

Secondary pancreatic tumors should be considered in those patients who present with a solitary pancreatic mass and who have had a previous non-pancreatic malignancy. Although ovarian metastasis to pancreas is rare, it has been reported in literature [14]. Differential diagnosis of a primary pancreatic neoplasm from a metastatic malignancy may be very difficult. Symptoms and signs are similar for both primary and secondary tumors, and radiologic imaging is unable to differentiate primary from secondary pancreatic lesions [15]. When a pancreatic mass is detected, CT scan is usually performed to evaluate the extent of disease. Radiological studies are useful for determining size and resectability, but a biopsy for tissue confirmation is required to differentiate primary and secondary pancreatic tumors [9]. In this case, MRI revealed a cystic lesion, but it was not able to be identified by an EUS-guided FNA either prior to surgery or intraoperation fluid analysis.

Metastatic tumors to the pancreas are rare indications for pancreatic resection. Resection of metastatic tumors to the pancreas has been only occasionally reported, and its role in improving survival or quality of life of the patients is not clearly defined [1, 4]. Although the value of surgical resection is poorly documented, resection may be deemed appropriate in selected cases [1]. We performed distal pancreatectomy, because we could not identify the origin of tumor whether it was primary or metastasis and eventually it was considered as primary distal pancreas cancer.

In conclusion, pancreatic metastasis of ovarian papillary serous adenocarcinoma has to be kept in mind when a patient with pancreatic mass has a history of ovarian malignancy. Surgical approach may offer the chance of longer survival in pancreas metastasis. Since pancreas resection due to pancreatic metastasis of ovarian cancer rarely reported, we considered it was worthful to report this case.
